# Intestinal-Type Adenocarcinoma: Classification, Immunophenotype, Molecular Features and Differential Diagnosis

**DOI:** 10.1007/s12105-017-0800-7

**Published:** 2017-03-20

**Authors:** Ilmo Leivo

**Affiliations:** Department of Pathology and Forensic Medicine, University of Turku, Turku, Finland

**Keywords:** Intestinal-type adenocarcinoma, Sinonasal adenocarcinoma, Sinonasal nonintestinal adenocarcinoma, Head and neck adenocarcinoma, Immunohistochemistry, Molecular pathology, Wood dust exposure

## Abstract

Intestinal-type adenocarcinoma is the second most frequent sinonasal adenocarcinoma. High incidence of these tumors is seen among workers with occupational wood dust exposure, particularly of hardwood dusts. Intestinal-type adenocarcinoma has striking histomorphologic and immunophenotypic similarities with colorectal adenocarcinomas, but on the level of molecular pathologic mechanisms these tumors have their own specific features different from gastrointestinal tumors. This article provides an update on current histopathologic classification of intestinal-type adenocarcinomas, their immunophenotypic properties, recent advances in molecular pathologic features and differential diagnostic considerations.

Intestinal-type adenocarcinoma (ITAC) is the second most common type of sinonasal adenocarcinoma after adenoid cystic carcinoma. It is composed of subtypes described by Dr. Barnes that resemble carcinomas or adenomas of intestinal origin, and occasionally the normal intestinal mucosa [1, 2]. ITACs occur mostly in males with a wide age range and a mean of 50–64 years. ITACs are most frequently localized in the ethmoid sinus (40%), the nasal cavity (25%) and the maxillary antrum (20%). Rare tumors with intestinal-type differentiation may also occur in other areas of the upper airways [3, 4], and in lung [5]. ITACs are aggressive malignancies with frequent local spread to the orbit, the skull base and the intracranial space, and with a possibility of metastatic spread.

Occurrence of ITAC has a strong association with occupational exposure to hardwood dusts [6–9]. In woodworking industries, workers with long-term exposure to hardwood dusts have an incidence approaching 1000 times that in control populations. Occupational wood dust exposure has been documented in ca. 20% of cases of ITAC, while the rest are sporadic. The highest incidences are seen in furniture industry using hardwoods, particularly beech and oak [8, 9]. The incidence of ITAC is also high among woodworkers who lay hardwood floors. Other occupational dust exposures with risk for ITAC have been reported in shoe and leather industry and in textile manufacture. Also long-term exposure to chromium and nickel has been incriminated [10]. The carcinogenic compounds in occupational dusts have not been identified, but etiologic roles for tannins or chronic inflammation have been speculated [10]. Dr. Barnes reported that patients with ITAC had cumulative exposure times for wood dusts of 40–43 years [1]. Furthermore, ITACs associated with dust exposure were diagnosed mostly in men (85–95%) and predominantly in the ethmoid sinus [1]. This contrasts with sporadic ITACs that are more frequent in women and often arise in the maxillary antrum.

## Classification

ITACs mimic the appearances of neoplastic large and small intestinal mucosa, and occasionally normal intestinal mucosa. Based on histopathologic parameters, Dr. Barnes classified ITACs into five categories: papillary, colonic, solid, mucinous, and mixed subtypes [1]. On the other hand, the classification of Kleinsasser and Schroeder [11] subdivided ITACs into papillary-tubular cylindrical cell type (corresponding to papillary, colonic, and solid subtypes), alveolar goblet cell type and signet-ring cell type (both corresponding to mucinous subtype), and transitional type (corresponding to mixed subtype). The histologic subtypes have been reported to correlate with differences in clinical behavior [1, 10, 11].

**Fig. 1:**
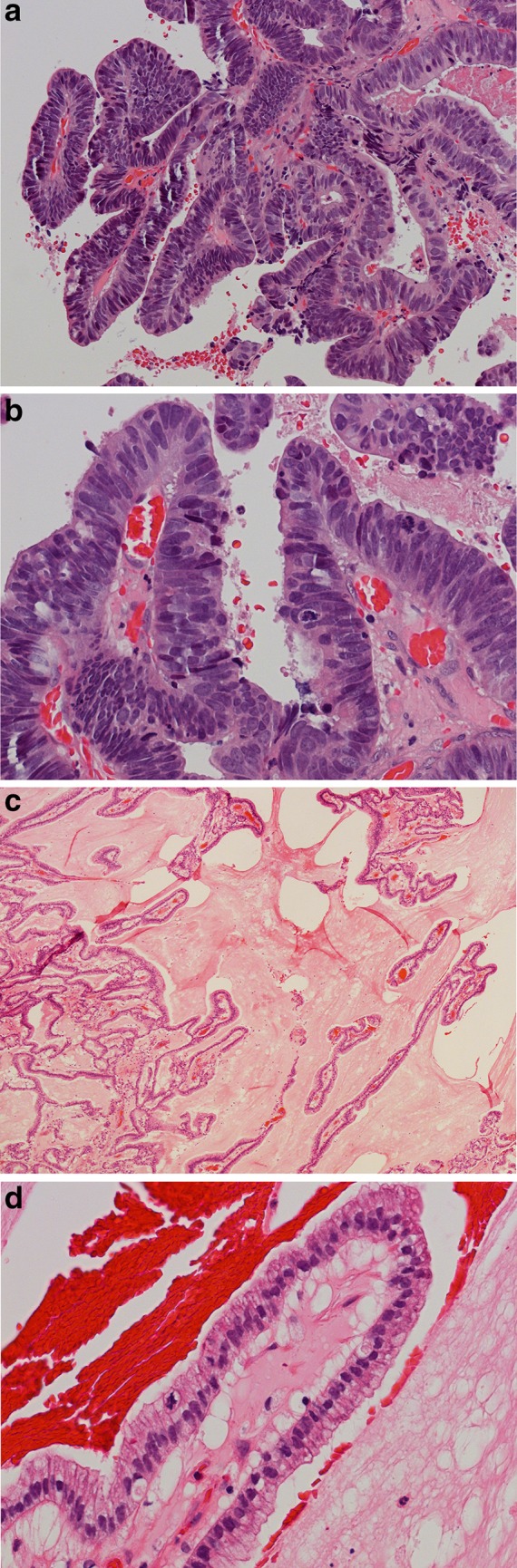
**a** Intestinal-type adenocarcinoma, papillary subtype. The tumor has ample papillary projections, and some glandular and tubular areas. H–E stain ×250. **b** The cells are usually cylindrical with elongated and pleomorphic hyperchromatic nuclei and nuclear crowding. Several mitotic figures are seen. H–E stain ×400. **c** Intestinal-type adenocarcinoma, highly differentiated papillary subtype resembling normal intestinal mucosa. H–E stain ×150. **d** Note the orderly arrangement of cylindrical cells in the papillae with only mild nuclear atypia. Occasional mitotic figures are seen. H–E stain ×400

In the Barnes classification papillary subtype of ITAC (ca. 18% of all) shows prominent papillary fronds with minor amounts of tubular structures (Fig. 1). ITACs of this type usually contain columnar goblet cells and they often resemble intestinal villous or tubular adenomas. Rarely, papillary ITACs may recapitulate the morphology of the normal intestinal mucosa with nearly normal-looking villi including the specialized cell types (goblet, resorptive, Paneth, and argentaffin cells) and the muscularis mucosae [12].

**Fig. 2:**
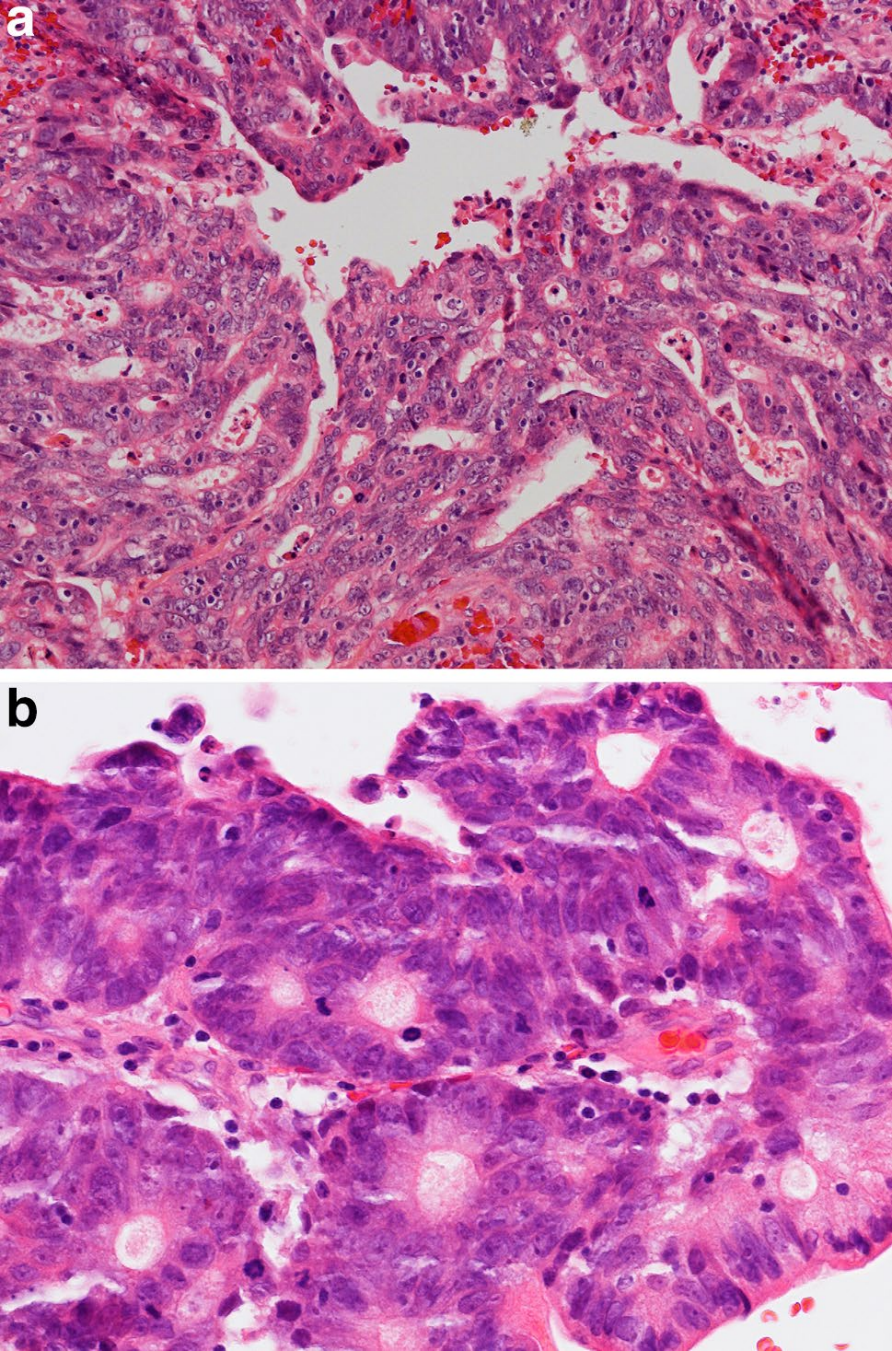
**a** Intestinal-type adenocarcinoma, colonic subtype. The tumor has glandular and trabecular areas resembling appearances of colorectal adenocarcinoma. H–E stain ×250. **b** The nuclei are crowded and highly pleomorphic and show some hyperchromasia. There is high mitotic activity. H–E stain ×400

**Fig. 3:**
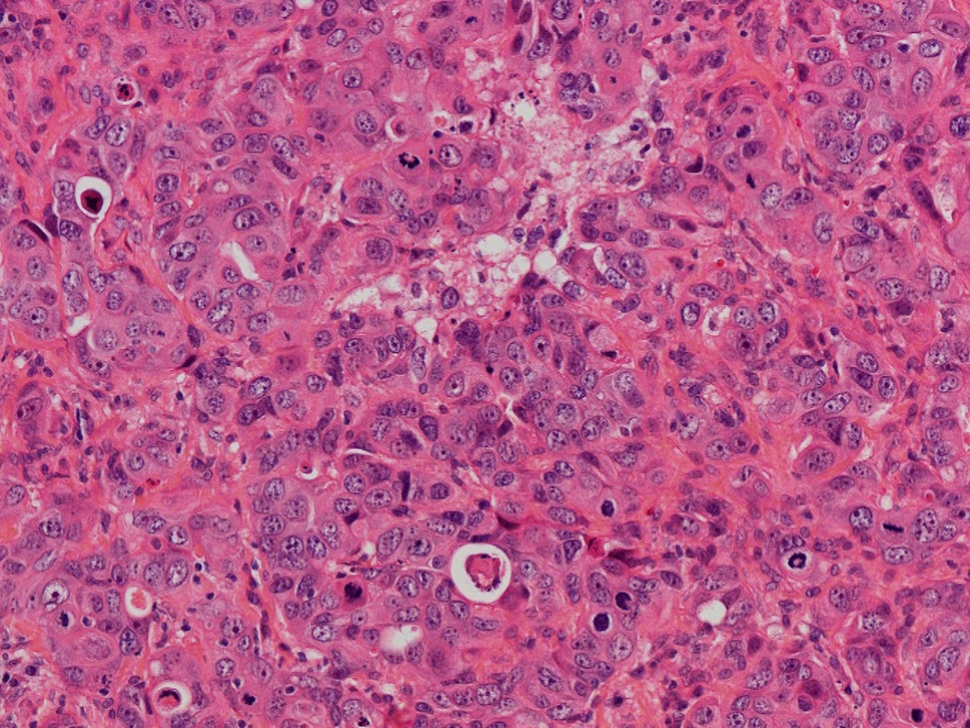
Intestinal-type adenocarcinoma, solid subtype. The tumor displays a diffuse growth pattern with minor amounts of poorly differentiated glandular lumina. There is high mitotic activity. H–E stain ×250

**Fig. 4:**
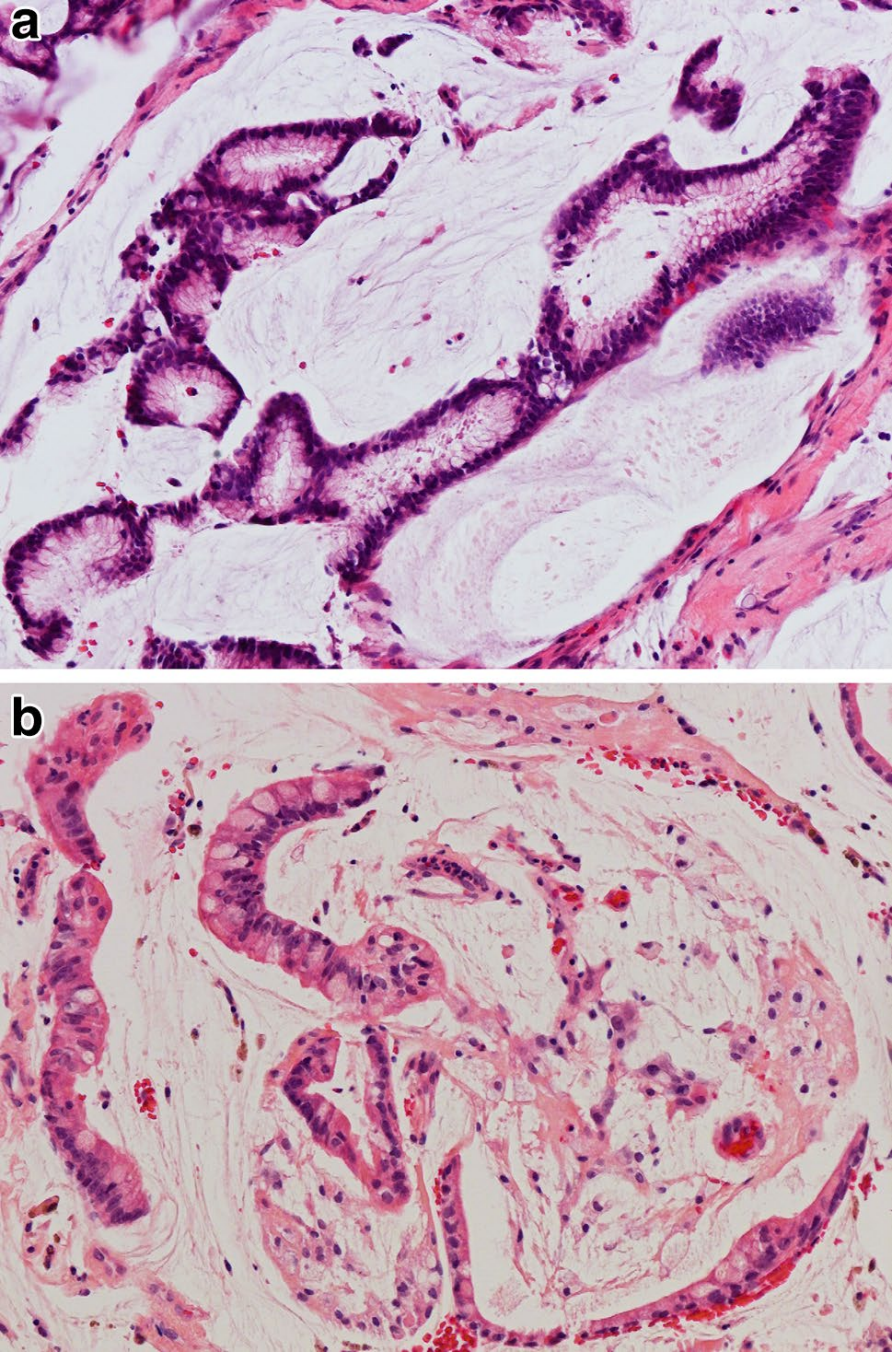
Intestinal-type adenocarcinoma, mucinous subtype. **a** Tumor cells form clusters with glandular lumina, **b** and strips with goblet-type cells, and are surrounded by ample pools of mucin. H–E stain ×400

Colonic subtype of ITAC is the most frequent (ca. 40%), displaying a glandular, tubular and trabecular architecture with few papillae. This tumor often mimics a conventional colorectal adenocarcinoma (Fig. 2). Columnar tumor cells are crowded back-to-back and they display nuclear pleomorphism. Intra- and extracellular mucins and few goblet cells may be seen. ITACs of the colonic subtype often show extensive invasive growth. Solid subtype of ITAC is less differentiated and features predominantly solid growth patterns with minor amounts of glandular structures (Fig. 3). Mucinous subtype of ITAC displays distended glands or cell clusters within pools of extracellular mucin (Fig. 4). Cells of signet-ring type may be seen. Mucinous ITACs resemble the mucinous variant of colorectal adenocarcinoma. Mixed subtype of ITAC contains different compositions of the various growth patterns above.

Exceedingly rare primary non-sinonasal ITACs of the head and neck have been reported in the base of the tongue, the major salivary glands, the pharynx and the larynx [3, 4, 13]. These malignancies with intestinal differentiation display heterogeneous microscopic appearances composed of glands and tubules reminiscent of colorectal adenocarcinomas. Positive staining of tumor cells for CK20, villin and/or CDX-2 attests for intestinal differentiation.

## Immunophenotype and Molecular Features

**Fig. 5:**
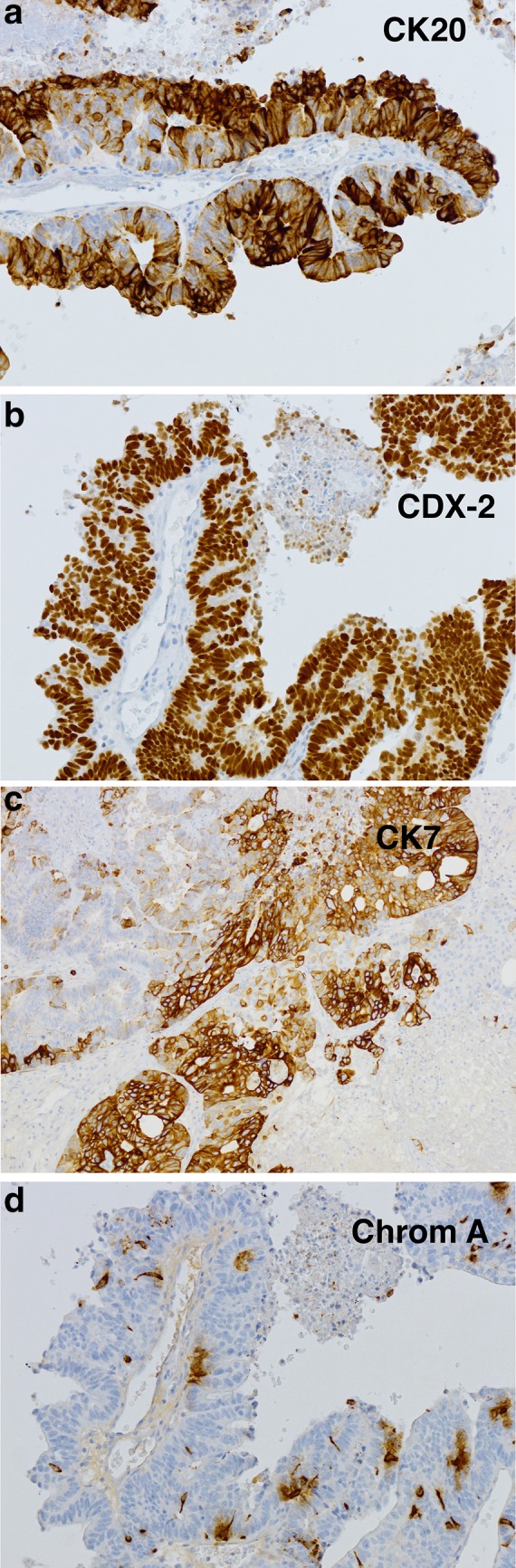
Immunohistochemical staining of intestinal-type adenocarcinoma, papillary subtype. **a** CK20 is seen in most tumor cells, **b** CDX-2 stains all tumor nuclei, **c** CK7 is variably positive in tumor cells, **d** chromogranin A is seen in occasional neuroendocrine cells. Peroxidase conjugated ABC Kit (Dako) ×400

Immunohistochemical staining indicates that ITACs are positive for CK20 (Fig. 5a), CDX-2 (Fig. 5b), villin, and MUC2, and variably positive for CK7 (Fig. 5c) [14–16]. Recently, the intestinal transcription and epigenetic factor SATB-2 has been identified as an additional marker of intestinal differentiation in ITAC [17]. Occasional neuroendocrine cells in ITACs often express chromogranin A (Fig. 5d) and/or synaptophysin. High levels of EGFR protein expression has been found in a subset of ITACs, mostly in woodworkers [18, 19]. ITACs have shown a normal expression of mismatch repair proteins, β-catenin and E-cadherin [20]. Although immunohistochemical studies have indicated close similarities between ITACs and colorectal adenocarcinomas, molecular pathological studies have revealed important differences. In contrast to colorectal carcinomas, activating mutations in *KRAS* and *BRAF* oncogenes are rare in ITAC [19, 21–23]. Overexpression of MET protein without *MET* gene amplification is frequent in ITAC, but not in intestinal carcinomas, and may provide opportunities for targeted therapies [24]. Loss of annexin A1 expression and diminished A2 expression has been reported in ITAC [25]. The frequency of *TP53* mutations in ITAC has ranged between 18–53% in different series [21, 26, 27]. The risk of such mutations increases with the duration and cumulative level of wood dust exposure [26, 27], but apparently not of smoking [27]. Nonsmokers have almost exclusively single missense-type *TP53* mutations with G>A transition, while smokers have less frequent and multiple frameshift mutations with G>T transition [27]. It has been speculated that mutations occurring during wood dust exposure might be related to reactive oxygen and/or nitrogen species generated by chronic inflammation [26, 27].

## Differential Diagnosis

The differential diagnosis of ITAC includes metastatic gastrointestinal carcinoma and sinonasal low-grade nonintestinal adenocarcinoma [1, 28]. A colorectal adenocarcinoma metastatic to the sinonasal tract cannot be distinguished from a primary sinonasal ITAC by any of the above immunohistochemical markers. Both ITACs and colorectal carcinomas express CK20, CDX-2, villin and MUC2, but the expression of CK7 in a tumor may be suggestive of ITAC. It is noteworthy that the expression of CK20 is more specific for ITAC than that of CDX-2. Additional specificity for ITAC can be obtained by staining for SATB-2 [17]. While CDX-2 is helpful when diagnosing ITAC, it is not fully specific as it can sometimes be expressed in sinonasal undifferentiated carcinomas and in salivary-type sinonasal adenocarcinomas [29]. Thus, if an intestinal-type tumor has been detected in the sinonasal tract, colonoscopy or colorectal radiographic studies should be performed to rule out primary colorectal adenocarcinoma. However, sinonasal metastases from gastrointestinal carcinomas are rare. In a review of 82 metastatic sinonasal malignancies, only five were derived from a primary tumor in the gastrointestinal tract [12]. Finally, the differential diagnosis of ITAC from sinonasal nonintestinal adenocarcinomas is supported by immunohistochemistry for CK20, CDX-2, villin and SATB-2 which only stain ITACs.

The treatment of ITAC is surgical resection varying from lateral rhinotomy to partial maxillectomy and total maxillectomy, with or without radiotherapy.

ITACs behave as high-grade malignancies. In 213 ITACs reviewed by Dr. Barnes, 50% of the patients developed local recurrences, 8% displayed cervical lymph node metastases, and 13% had distant metastases. A total of 60% of patients died of disease. ITACs associated with wood dust exposure had a somewhat better prognosis (with 50% survival rates at 5 years) than sporadic ITACs (with 20–40% survival rates at 5 years). Well-differentiated papillary ITACs pursue an indolent course, but solid and mucinous subtypes have poorer outcomes [1, 10, 11].
